# Newborn Screening for Spinal Muscular Atrophy in the Republic of Moldova: A Feasibility Study and First Steps

**DOI:** 10.3390/ijns12020038

**Published:** 2026-05-28

**Authors:** Iulia Coliban, Natalia Usurelu, Igor Opalco, Sergiu Gladun, Victoria Sacara

**Affiliations:** 1 Human Molecular Genetics Laboratory, Institute of Mother and Child, MD-2062 Chisinau, Moldova; victoriasacara@hotmail.com; 2 Laboratory of Prevention of Hereditary Pathologies, Institute of Mother and Child, MD-2062 Chisinau, Moldova; natalia.usurelu@yahoo.com; 3 Center for Human Reproduction and Medical Genetics, Institute of Mother and Child, MD-2062 Chisinau, Moldova; dropalco@gmail.com; 4 Institute of Mother and Child, MD-2062 Chisinau, Moldova; gladunsergiu@gmail.com

**Keywords:** neonatal genetic screening, spinal muscular atrophy, pilot program implementation, rare disease management, public health

## Abstract

Spinal muscular atrophy (SMA) is a severe neuromuscular disorder in which presymptomatic treatment substantially improves survival and motor outcomes, yet newborn screening for SMA remains unevenly implemented across Europe, and evidence from lower-resource health systems is needed to guide scale-up. In this study, we assessed the feasibility, diagnostic performance, and public health implications of implementing neonatal SMA screening in the Republic of Moldova within an established national newborn screening framework. A pilot genetic screening program was conducted using dried blood spot (DBS) samples collected through routine newborn screening workflows; *SMN1* exon 7 deletion testing was performed by real-time polymerase chain reaction (qPCR), and positive findings were confirmed by multiplex ligation-dependent probe amplification (MLPA), alongside the evaluation of operational integration and system-level requirements. Screening was operationally feasible within existing DBS processes and demonstrated high analytical performance, consistent with published international experience, although performance results should be interpreted cautiously due to the limited sample size. Two SMA cases were confirmed in a small cohort, enabling early diagnosis and timely referral for disease-modifying therapy, and integration into the existing program was practical and resource-efficient. These findings support the incorporation of SMA into national newborn screening panels using DBS-based molecular methods, highlighting an implementable model for introducing advanced genetic testing within routine public health services.

## 1. Introduction

Spinal muscular atrophy (SMA) (OMIM: 253300) is an autosomal recessive hereditary disorder with an incidence of approximately 1 in 10,000 newborns, classifying it as a rare disease [1]. It is caused by mutations that lead to the progressive and irreversible degeneration of motor neurons, resulting in muscle weakness and atrophy [2,3]. The *SMN1* gene, located on chromosome 5q13.2, encodes the survival motor neuron protein, which is essential for motor neuron function. Homozygous deletion of exon 7 in *SMN1* is strongly associated with the development of SMA [4,5]. Low SMN protein levels lead to loss of motor neuron function in the spinal cord, ultimately causing skeletal muscle degeneration [6].

Diagnosis of SMA relies exclusively on molecular genetic testing, as no biochemical markers are available [7,8]. Currently, testing is most often initiated only after symptom onset, following a genetic consultation. Because clinical presentation is heterogeneous and early signs—such as hypotonia—can be nonspecific, diagnosis is frequently delayed, reducing the effectiveness of therapeutic interventions [9,10,11].

Until 2016, no treatment options existed for SMA; however, since 2017, several innovative therapies have become available, including gene therapy and *SMN2*-modulating drugs [2,12]. *SMN2* is a pseudogene structurally like *SMN1* but produces only 10% of functional protein. Its copy number is a key modifier of disease severity: fewer *SMN2* copies are generally associated with earlier-onset and more severe phenotypes, particularly SMA type I, which carries the highest mortality, whereas a higher *SMN2* copy number is linked to milder type II–IV phenotypes and improved survival. Therefore, early initiation of SMN-restoring therapies is especially critical in patients with a low *SMN2* copy number, in whom rapid disease progression may otherwise lead to irreversible motor neuron loss, severe disability, and increased mortality [13]. With no biochemical marker available to diagnose this disease, neonatal genetic screening has become increasingly urgent, as early identification enables rapid treatment initiation before clinical symptoms appear [1,9,14,15].

The Republic of Moldova is a lower-middle-income country in Eastern Europe, with a population of approximately 2.4 million inhabitants and a nominal GDP of around USD 19 billion. In 2024, the GDP per capita was estimated at EUR 7037. During the same year, 23,862 newborns were registered (excluding Transnistria), of whom 23,108 were covered by the national newborn screening program, corresponding to an estimated coverage rate of approximately 99%. Despite this near-universal population coverage, the national screening program remains limited in scope, as it currently includes only phenylketonuria (PKU). Screening is performed through a single national screening center, while molecular genetic diagnostics for rare diseases are available through only one specialized molecular genetics laboratory in the country, located at the Institute of Mother and Child (IMC). Notably, the cost of screening per newborn was reported at EUR 0.65, representing the lowest cost among the countries analyzed [16]. Within this constrained diagnostic landscape, epidemiological data remains essential for understanding the burden of rare genetic disorders. In the Republic of Moldova, earlier national data on hereditary neuromuscular diseases (1991–2018) documented SMA as part of the “core” conditions, with a reported population prevalence of 8.43 ± 0.15 per 100,000 [17,18], specifying that deletions of exon 7 and exon 8 of the *SMN1* gene were detected in approximately 85% of patients with SMA types I–III [17]. More recent local data (from the Human Molecular Genetics Laboratory (HMGL) of IMC) identified 33 newly diagnosed SMA cases during 2018–2025 [19,20,21].

In the era of expanding SMA therapies, early genetic diagnosis serves as a roadmap for timely and targeted intervention. In this context, integrating neonatal genetic screening into public health systems is of major importance, as early detection of severe hereditary diseases like SMA improves health outcomes, reduces long-term healthcare costs, and supports timely access to effective therapies.

The aim of this study was to develop and implement a laboratory protocol for neonatal genetic screening of SMA, covering the entire workflow from sample collection to the communication of the result to the family. This integrated approach is based on a pilot study in a PhD program conducted at the IMC between 2022 and 2024 and seeks to ensure a standardized, efficient, and safe workflow aligned with national and international neonatal screening requirements. Such a protocol enables the rapid and accurate identification of affected newborns, supports timely treatment strategies, and contributes to improving the quality of life of patients with SMA—an objective of major relevance to public health management.

## 2. Materials and Methods

The NBS program for SMA involves collaboration between multiple stakeholders from the policy, diagnostic, and healthcare systems. Methodologies that enhance interdisciplinary learning, leading to multidisciplinary, patient-centered growth, are essential in program application and effectiveness [14,22,23]. Among the first steps taken in this direction was the development and approval of the national clinical protocol for SMA (Atrofia Musculară Spinală, PCN-402, ed.1, approved by the Health Ministry, nr. 417, on 5 May 2022) [24,25,26]. Moreover, at the governmental level, negotiations are ongoing for the approval of the prices proposed by the holders of marketing authorizations for treatments with international trade names, so that the insured can benefit from the treatments included on the list.

Thus, within the tertiary-level scientific and practical Institute of Mother and Child and the Human Molecular Genetics Laboratory (HMGL), in collaboration with the “N. Testemițanu” State University of Medicine and Pharmacy, an initiative was developed for the presymptomatic diagnosis of SMA-associated mutations in newborns from the Institute’s maternity wards whose parents or legal representatives agreed to participate in the study. This study was initiated within a small research project that is focused only on staff salary and reagents, not infrastructure development, named “Genomic medicine and metabolomic research in the service of the prevention of genetic diseases for healthy generations in the Republic of Moldova ”(Acronym: SCREENGEN, code: 20.80009.8007.22, for 2020–2023), which continues within the institutional subprogram “Diagnosis and monitoring of genetic diseases in the prevention of maternal and child health” (Acronym: DiMoGEN, code 140102, 2024–2027) in a PhD program study. The initiative consists of developing a system for testing newborns for mutations in genes associated with SMA to support implementation of a pilot genetic screening program for the presymptomatic diagnosis of SMA. **Participants.** The subjects were newborns from the maternity ward of the Institute of Mother and Child (IMC), where a dried blood spot card collection system is implemented.

Inclusion criteria:

- Newborns aged 24–72 h of life;
- Gender: male and female;
- Children born in the IMC, Chisinau, Republic of Moldova;
- Written informed consent obtained from the parents or legal representatives of all newborns prior to inclusion in the study.

Exclusion criteria:

- Children whose parents or legal representatives did not sign the Consent and Informed Agreement;
- Children whose legal representatives refused to participate;
- Newborns with acute illness or congenital anomalies in the neonatal intensive care unit;
- Cards with dried blood spots collected incorrectly;
- Samples with concentrations of less than 20 ng/mL of genomic DNA obtained from spots on filter paper.

**Methods.** The processing of samples within the neonatal screening protocol for SMA is essential to ensuring a rapid and accurate diagnosis. Every step, from the initial collection to the communication of the result, must comply with strict quality and traceability standards, thereby guaranteeing the accuracy of testing and early intervention in positive cases.

- *Sample collection.* Blood samples were collected 48–72 h after birth using Guthrie cards (DBSs) in the neonatal units of the IMC. Samples were prepared according to international standards and stored at +4 to +8 °C after drying. Each sample was accompanied by a newborn registration form containing maternal information, infant sex, and anthropometric and demographic data, together with written informed consent from parents or legal representatives (Form S1). All study procedures complied with the ethical principles of the Declaration of Helsinki, including its 2013 revision [27]. DBS samples were transported to the HMGL using the same logistics pathway as the national phenylketonuria screening program.
- *Sample registration*. Upon arrival at the laboratory, DBS cards were checked for integrity and registered in a digital sample-management file. Each sample was assigned a unique identification code to ensure full traceability throughout the analytical process. DBS cards were stored at +4 to +8 °C until analysis. A primary punch was obtained from the DBS for routine PKU screening, and subsequent punches from the same card were used for SMA testing.
- *Sample processing*. Since SMA lacks a biochemical marker, diagnosis relies on detecting the presence or absence of exon 7 in the *SMN1* gene. Genomic DNA was extracted from a 3.2 mm DBS punch using column-based nucleic acid purification method with the GeneJET Whole Blood Genomic DNA Purification Kit (Thermo Fisher Scientific, Ref. No. K0781, Vilnius, Lithuania) [28]. The procedure involves excision of the DBS filter section and rehydration in 200 μL of 1× PBS for 5–10 min at room temperature, followed by proteinase K lysis, protein precipitation with ethanol, column purification through washing steps, and final DNA elution. This column-based method is rapid and provides high-quality DNA suitable for molecular testing. After DNA extraction, sample concentration was measured using a Qubit 3.0 fluorometer with the Qubit dsDNA HS Assay Kit (Invitrogen, Thermo Fisher Scientific Inc., Carlsbad, CA, USA) according to the manufacturer’s protocol, using the provided working solution and reference standards.
- *Molecular genetic screening and testing*. Following quantification, DNA samples were diluted with molecular biology-grade deionized H_2_O (Fermentas, Vilnius, Lithuania) to a final concentration of 20 ng/μL using the C_1_V_1_ = C_2_V_2_ formula, where C represents concentration and V represents volume. Real-time PCR was performed to detect the presence or absence of exon 7 in the *SMN1* gene, using exon 12 of the *ALB* gene as an internal amplification and quality-control target. The workflow included real-time amplification, analysis of amplification and melt curves, software-based result evaluation, and final interpretation by the operator. The SMA molecular genetic test used in this study was developed and optimized within the HMGL using authorized access to the institutional biobank (agreement from 18 September 2020). The assay was based on real-time qPCR followed by high-resolution melting (HRM) analysis using SYBR Green intercalating dye. Custom primers were designed to amplify the *SMN1* exon 7 target region containing sequence differences that allow for discrimination between *SMN1* and *SMN2*, while *ALB* exon 12 served as an internal amplification control. Primer sets for *SMN1* exon 7 were custom-developed using Primer-BLAST (Primer3 version 2.5.0; National Center for Biotechnology Information, Bethesda, MD, USA), synthesized by Invitrogen (Invitrogen/Thermo Fisher Scientific, Carlsbad, CA, USA), supplied in lyophilized form, reconstituted in nuclease-free water, and adjusted to a working concentration of 10 pmol/μL before use.

The *SMN1* exon 7 primer sequences were as follows: forward primer, 5′-TTTTATTTTCCTTACAGGGTTTCA-3′; reverse primer, 5′-GTTTTACATTAACCTTTCAACTTTTT-3′. Primers targeting *ALB* exon 12 were selected from previously described sequences and were used as an internal amplification control under the same qPCR-HRM cycling conditions: forward primer, 5′-AGCTATCCGTGGTCCTGAAC-3′; reverse primer, 5′-TTCTCAGAAAGTGTGCATATATCT-3′. The assay was developed and optimized at the HMGL and approved as part of Innovation Act No. 562 from 07 March 2024. The assay was validated using 25 reference DNA samples, including 15 SMA-negative and 10 SMA-positive samples with confirmed *SMN1* exon 7 deletion. In this validation set, the assay demonstrated complete concordance with the expected results and subsequently served as the basis for a pilot screening study using 346 newborn DBSs.

The method integrates qPCR with HRM analysis, enabling accurate detection of the pathogenic *SMN1* exon 7 deletion using a custom HMGL-developed workflow. The qPCR-HRM assay was performed on a 7500 Real-Time PCR System (SN: 275009565; Applied Biosystems, Carlsbad, CA, USA) using the instrument-associated software for real-time fluorescence acquisition, amplification plot generation, melting curve analysis, and result interpretation. The run was configured using the SYBR Green chemistry workflow, with melt curve analysis enabled. Each 20 μL reaction contained 7 μL molecular biology-grade deionized H^2^O (Fermentas, Vilnius, Lithuania), 10 μL PowerTrack™ SYBR Green Master Mix, [2X] (Ref. No. A46012; Applied Biosystems by Thermo Fisher Scientific, Vilnius, Lithuania), 0.5 μL forward primer [10 pmol/µL], 0.5 μL reverse primer [10 pmol/µL], and 2 μL genomic DNA [20 ng/μL]. The amplification protocol consisted of initial polymerase activation and DNA denaturation at 95 °C for 5 min, followed by 40 cycles of denaturation at 95 °C for 15 s and primer annealing/extension at 59 °C for 1 min. HRM analysis was subsequently performed using the following melting protocol: 95 °C for 15 s, 60 °C for 1 min, 95 °C for 30 s, and 60 °C for 15 s.

All procedures adhered to approved regulations for genetic testing and biobank usage (approval No. 01-13/591, 18 September 2020). In the analysis of positive neonatal SMA screening results, MLPA (multiplex ligation-dependent probe amplification; SALSA MLPA Probemix P021 SMA kit, Lot B1641; MRC Holland, Amsterdam, The Netherlands) [29] was used as the confirmatory method first implemented in Moldova in 2022 (Implementation Act No. 483 from 17 March 2022). This method served as the diagnostic gold standard and was essential in reducing the risk of false-positive results, due to its ability to accurately determine *SMN1* and *SMN2* copy number and to identify both homozygous and heterozygous *SMN1* deletions or gene conversions.

To complement the initial analytical validation of the custom HMGL qPCR-HRM assay, a subset of 75 samples was additionally tested using a commercially available real-time PCR kit, the NeoNat SCID-SMA Multiplex Real-Time PCR Kit (Labsystems Diagnostics Oy, Vantaa, Finland; Ref. No. 8100411), targeting SMA-associated genes. This comparative analysis was performed to assess the concordance of the locally developed assay with a commercially available method and to evaluate its feasibility as a cost-effective and locally implementable alternative for neonatal SMA screening, particularly in settings where access to commercial kits may be limited by financial, logistical, or supply constraints. The commercial assay was performed according to the manufacturer’s instructions, and results were compared with those obtained using the custom HMGL qPCR-HRM method.

- *Quality control and run validity criteria*. For the custom HMGL qPCR-HRM assay, each run included a no-template control, a positive control DNA sample with confirmed homozygous *SMN1* exon 7 deletion, and a negative control DNA sample with normal *SMN1* status. Each sample was analyzed using three reactions: two reactions targeting *SMN1* and one reaction targeting the internal control *ALB*. A qPCR run was considered valid only if the no-template control showed no amplification, the positive and negative controls produced the expected amplification and melting curve profiles, and the *ALB* internal control amplified appropriately in all analyzed samples. Samples with absent or inadequate *ALB* amplification, atypical amplification curves, or inconclusive melting profiles were repeated.

For the commercial NeoNat SCID-SMA Multiplex Real-Time PCR assay, run validity and quality-control interpretation were performed according to the manufacturer’s instructions. Commercial assay results were accepted only when the internal control and assay-specific controls fulfilled the validity criteria specified by the manufacturer.

- *Data analysis*. This study was designed as an observational cohort. Sample size was estimated using Epi Info software, version 7.2.2.6, StatCalc “Sample Size and Power” module, based on a 99.0% confidence interval, 80.0% statistical power, an expected qPCR-based SMA screening performance of approximately 98.0%, and an adjusted 10.0% non-response rate, resulting in a required sample size of at least 172 newborns.

First-tier qPCR-HRM screening data generated on a real-time PCR system were analyzed using 7500 Software v2.3. Amplification plots and HRM profiles were interpreted by comparison with control profiles. The *ALB* exon 12 internal control was used to confirm adequate amplification and sample quality, showing an expected melting peak at approximately 82–83 °C. The *SMN1* exon 7 amplicon showed an expected melting peak at approximately 75–76 °C in samples without *SMN1* exon 7 deletion. Samples with deletion-suggestive profiles showed an absence or alteration of the expected *SMN1* melting peak, sometimes accompanied by a broad lower-temperature signal around 71–72 °C. This lower-temperature signal was interpreted only in the context of valid control results, appropriate *ALB* amplification, and comparison with positive and negative control profiles.

Screen-positive samples were confirmed by multiplex ligation-dependent probe amplification using the SALSA MLPA Probemix P021 SMA kit (P021-100R; Lot B1641; MRC Holland, Amsterdam, The Netherlands). MLPA results were analyzed using Coffalyser.Net software, version v.240129.1959, together with the corresponding lot-specific Coffalyser sheet. All data were securely stored and checked for accuracy before final interpretation.

- *Cost-efficiency assessment*. The cost-efficiency assessment was performed based on the assay layout and workflow characteristics of the custom HMGL method and the commercial kit-based approach. For the HMGL method, the layout allowed for the analysis of 32 samples per 96-well plate, and the complete workflow was included in the assessment. The cost per tested sample was calculated considering reagent and consumable use, number of reactions per sample, plate capacity, and workflow organization. The same parameters were evaluated for the commercial kit-based approach according to the manufacturer’s instructions.
- *Confidentiality*. This study follows the Declaration of Helsinki and WMA regulations. Participant data will be de-identified and stored in the HMGL database, with re-contact allowed only when clinically necessary.

## 3. Results

The proposed method was integrated into a molecular-genetic screening process for the early identification of SMA as part of a pilot study conducted at the IMC Maternity Hospital between July 2022 and March 2024. During this period, approximately 7000 births occurred, of which 346 newborns were enrolled, corresponding to a participation rate of approximately 5%. Accordingly, 346 informed consents were obtained, and dried blood spot samples collected on Guthrie cards were processed.

**Figure 1:**
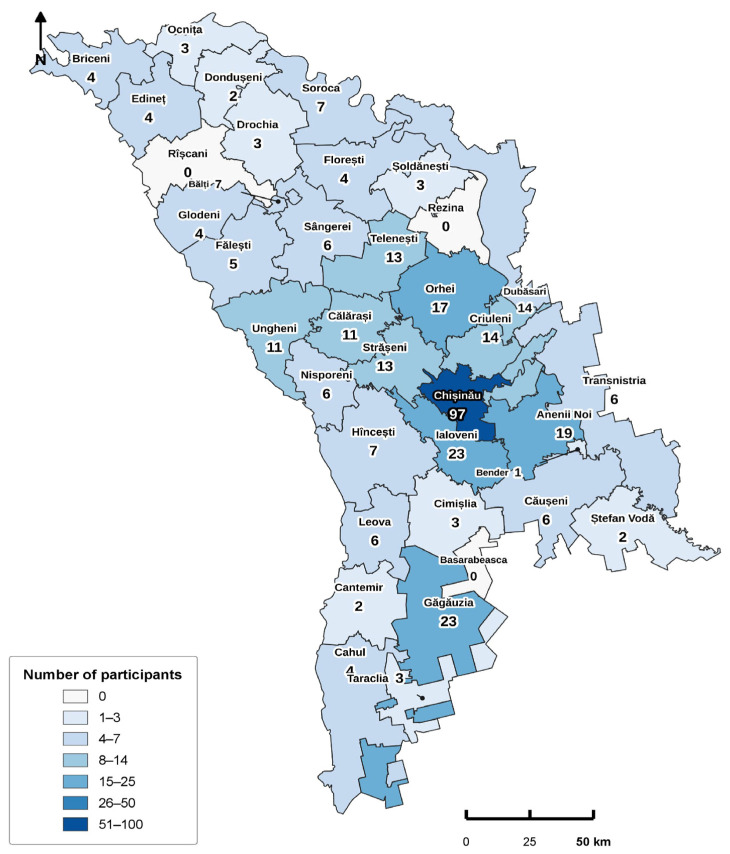
Geographic distribution of study participants collected at the IMC tertiary referral maternity ward by region of origin in the Republic of Moldova. For mapping purposes, Stăuceni was aggregated with Chișinău, Comrat and Ceadîr-Lunga with Găgăuzia, and Rîbnița and Slobozia with Transnistria.

Figure 1 illustrates the geographic distribution of DBS samples collected from newborns delivered at a level III perinatal referral center at the IMC, which receives pregnant women from across the country. A total of 346 samples were obtained from participants originating from 38 districts and municipalities, highlighting the geographic heterogeneity of the study population. The largest proportions originated from Chișinău (93/346, 26.9%, shown as 97 in Figure 1 after aggregation with Stăuceni for mapping purposes), Ialoveni and Gagauzia (23/346, 6.6% each), Anenii Noi (19/346, 5.5%), Orhei (17/346, 4.9%), and Criuleni and Dubăsari (14/346, 4.0% each). The median number of samples per region was approximately six, while four regions contributed only one sample. This distribution reflects the wide national catchment area and referral role of the maternity hospital.

Importantly, the study site is located within the same institution that hosts the national neonatal screening point of phenylketonuria, the Medical Genetics and Reproductive Health Center of IMC, and the genetic laboratory providing specialized genetic counseling support. This institutional context supports the feasibility of a future expansion of neonatal screening activities, including molecular genetic screening approaches.

### 3.1. Assessment of Screening Test Sensitivity for Exon 7 Deletion in SMN1 and Result Validation

**Figure 2:**
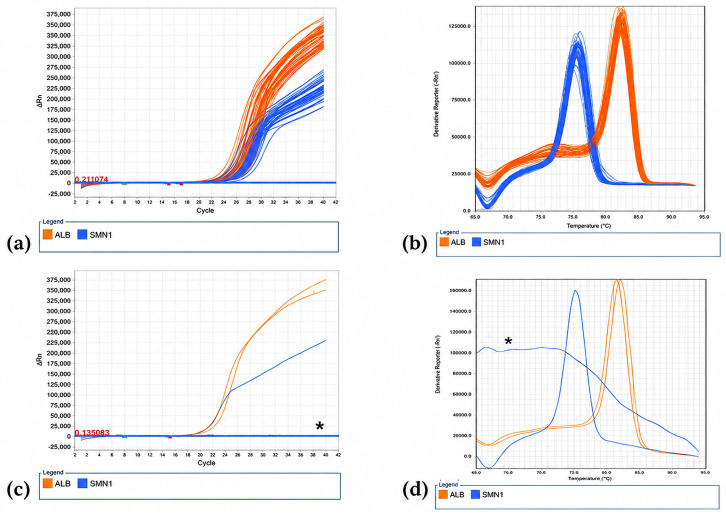
qPCR-HRM screening profiles for *SMN1* exon 7 and the *ALB* internal control in the screened cohort. (**a**) Representative qPCR amplification curves of SMA screen-negative samples, showing amplification of *SMN1* exon 7 target locus and *ALB* exon 12 internal control. (**b**) Corresponding HRM curves of the same SMA screen-negative samples, showing the expected melting profiles for *SMN1* exon 7 and *ALB* exon 12. (**c**) Representative qPCR amplification curves of an SMA screen-positive sample shown together with an SMA screen-negative sample for comparison. The SMA screen-positive sample shows the absence/deletion of the *SMN1* exon 7 target signal with preserved amplification of the *ALB* internal control. (**d**) Corresponding HRM curves of the same SMA screen-positive and SMA screen-negative samples, showing the deletion-associated *SMN1* profile in the screen-positive sample and preserved *ALB* control amplification. Blue curves indicate the *SMN1* target locus, and orange curves indicate the *ALB* internal control locus. Asterisks indicate the SMA screen-positive samples showing an absence of specific *SMN1* exon 7 amplification (**c**) and the corresponding melting peak (**d**).

A total of 346 newborns were tested in this pilot study assessing the feasibility of neonatal genetic screening for this rare disorder. The qPCR-HRM custom screening test evaluated the amplification of *SMN1* exon 7, with *ALB* exon 12 used as an internal control. Representative qPCR amplification and HRM profiles are shown in Figure 2a–d.

Most samples showed the expected SMA screen-negative profile, with preserved amplification of both *SMN1* exon 7 and the *ALB* internal control. Two samples showed an absence of *SMN1* exon 7 amplification together with altered HRM profiles, while *ALB* amplification was preserved, indicating valid DNA amplification and supporting an SMA screen-positive result. Representative SMA screen-negative profiles are shown in Figure 2a,b, whereas representative SMA screen-positive profiles are shown in Figure 2c,d. The two screen-positive samples were subsequently subjected to confirmatory MLPA testing, as described in Section 3.3.4.

The custom qPCR-HRM assay detected all samples with confirmed homozygous *SMN1* exon 7 deletion included in the validation set, corresponding to a 100% detection rate for this specific mutation under the tested conditions. Since the assay targets homozygous *SMN1* exon 7 deletion, rare SMA-causing variants not detectable by this approach may be missed; therefore, the estimated clinical sensitivity for all SMA forms is expected to be approximately 95%. In the pilot cohort, all screen-positive samples were confirmed by MLPA, and no discordant results were observed. However, given the limited number of positive cases and the rarity of SMA, these findings should be interpreted as preliminary performance data and should not be considered population-level estimates of sensitivity, specificity, predictive values, or disease incidence, but at most a feasibility study of the application of this assay for SMA screening.

A subset of 75 samples was analyzed using both the custom HMGL qPCR-HRM assay and the commercial NeoNat SCID-SMA Multiplex Real-Time PCR Kit. The subset size was determined by the commercial kit plate layout, which allowed a maximum of 75 patient samples per 96-well plate after inclusion of the required controls. The comparative results are presented in Table 1. The results obtained with the custom HMGL assay were concordant with those obtained using the commercial assay, supporting the analytical validity of the developed screening method [30].

### 3.2. Evaluation of Cost and Workflow Efficiency

The comparative cost-efficiency analysis showed differences in 96-well plate utilization between the custom laboratory-developed method and the commercial multiplex assay when applied to the optimized neonatal screening workflow for SMA based on the workflow parameters and cost per tested sample described above.

For the custom HMGL assay, the requirement of three wells per sample, together with the allocation of wells for no-template, positive, and negative controls, resulted in an operational capacity of approximately 32 samples per 96-well plate. In contrast, the commercial kit, using the manufacturer-defined multiplex layout and assay-specific controls, allowed for testing of up to 75 samples per 96-well plate.

At maximum plate utilization, the estimated total cost per run was approximately EUR 155 for the HMGL method and EUR 381 for the commercial kit. This corresponded to an estimated cost per sample of approximately EUR 5.00–5.20 for HMGL and EUR 5.10 for the commercial kit. Therefore, when both methods are evaluated at maximum theoretical plate capacity, the per-sample cost is comparable, while the commercial kit offers a higher sample throughput per plate (Table 2). However, under lower or variable daily sample volumes, the HMGL method may offer greater workflow flexibility and be economically advantageous in routine pilot screening conditions.

Therefore, this analysis reflects real-world laboratory conditions during the pilot phase rather than maximum theoretical efficiency.

### 3.3. Result Reporting

#### 3.3.1. Rejected or Non-Identified Samples

If a DBS sample could not be processed because of inadequate sample quality, insufficient material, or other technical issues, the sample was rejected and the parents or medical representative were notified by a dedicated letter requesting repeat sample collection (Figure S1). Similarly, if the sample could not be identified according to the screening participant list or the samples delivered to the laboratory, a notification letter was issued requesting a new sample for neonatal screening (Figure S2).

#### 3.3.2. Negative Results

Negative screening results were recorded in the laboratory database but not reported to families.

#### 3.3.3. Positive Screening Results

For positive results, the DBS sample was retested; If the result was confirmed on retesting, a notification letter (Figure S3) was sent to the family and neonatologist, outlining the screening result and recommending urgent referral for confirmatory evaluation and genetic consultation.

#### 3.3.4. Confirmatory Testing

Following a positive SMA screening result, parents were promptly invited for clinical consultation. After clinical examination, peripheral blood was collected in EDTA tubes for second-tier confirmatory testing using MLPA technique. Its implementation in 2021 enabled, for the first time in the Republic of Moldova, SMN2 copy number quantification and patient stratification. Confirmatory testing established, in the first case, a homozygous deletion of SMN1 exon 7 with three SMN2 copies and a heterozygous deletion of NAIP exon 5. In the second case, MLPA confirmed a homozygous deletion of SMN1 exon 7 with one SMN2 copy and no NAIP exon 5 deletion [5]. After confirmatory results became available, parents were invited to a follow-up consultation to discuss the genetic diagnosis, receive genetic counseling, and determine therapeutic options.

### 3.4. Follow-Up of SMA-Positive Cases

**Figure 3:**
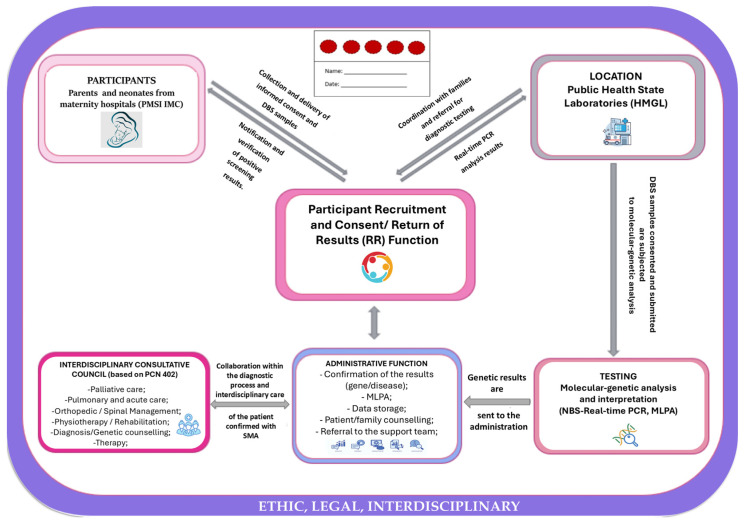
Proposed operational pathway of the newborn genetic screening program for SMA. Workflow of the neonatal genetic screening pathway for SMA, illustrating participant recruitment, DBS sample collection, laboratory molecular testing (PCR/MLPA), administrative validation and reporting, and interdisciplinary clinical follow-up within an ethical, legal, and coordinated public health framework. PCN—National Clinical Protocol; PMSI IMC—Public Medical–Sanitary Institute of Mother and Child; HGML—Human Molecular Genetics Laboratory; MLPA—Multiplex Ligation-dependent Probe Amplification; NBS—Newborn Screening; Real-Time PCR—real-time polymerase chain reaction.

Follow-up of SMA-positive cases follows National Clinical Protocol 402/2022, ensuring a clear pathway for early intervention through coordinated screening, molecular diagnosis, and interdisciplinary care (see the workflow in Figure 3).

## 4. Discussion

Although rare, with a globally estimated incidence of 1–6 cases per 10,000 live births, SMA remains an important hereditary neuromuscular condition; in the Republic of Moldova, it is further characterized by a reported population prevalence of 8.43 ± 0.15 per 100,000 inhabitants [31]. In our setting, 33 newly diagnosed cases (2018–2025) correspond to an observed birth incidence of approximately 1 in 7300 live births, based on an average of ~28,000 births per year [21]. Importantly, four of these infants, specifically, two born in 2019, one in 2020, and one in 2021, were born at the Institute of Mother and Child maternity ward, the site where the SMA newborn screening (NBS) project was later implemented. This situation highlights the need to strengthen molecular genetic diagnostics, including the introduction of quantitative methods for CNV assessment and the expansion of existing molecular capacities. In this context, the adoption of newborn screening for SMA represents a feasible public health intervention capable of translating early identification into timely confirmatory diagnosis and treatment access.

Even though 33 cases of SMA have been diagnosed in the past 7 years both clinically and genetically, with stratification into types enabled by the implementation of MLPA, the true disease burden may still be underestimated. In the Republic of Moldova, population decline from approximately 2.7 million in 2014 to 2.3–2.4 million in 2024, driven by migration and reduced birth rates, further complicates case ascertainment and follow-up [21]. In addition, cross-border healthcare-seeking behavior, with patients traveling abroad for treatment, may result in loss of valuable time and incomplete case registration. Diagnostic delays and population mobility support the need for newborn screening to enable early identification and maximize the benefits of presymptomatic therapy.

During the research implementation phase, the screening of 346 DBS samples identified two SMA cases, supporting detection capability and demonstrating practical feasibility within a single maternity setting. The clinical evaluation of the screen-positive cases included neurological assessment and confirmatory genetic testing by MLPA. One infant was followed longitudinally and managed according to national SMA protocols, with follow-up available up to 10 months of age. The second infant underwent initial clinical and genetic evaluation, presenting with hypotonia and motor development delay; however, follow-up data were limited, as the patient did not continue specialized medical care. Notably, neither infant received disease-modifying therapy for SMA in our country.

Although both SMA-positive cases identified in our NBS cohort originated from Chișinău, this likely reflects referral patterns and internal migration rather than true geographic clustering. Previous epidemiological data from the Republic of Moldova indicate that SMA cases are distributed across multiple regions, with a moderate correlation to population size and ethnic composition (Spearman ρ ≈ 0.49), but without significant regional clustering [17]. Given the small sample size (2/346) and the study setting in a tertiary referral maternity center serving a complex obstetric population, these findings should be interpreted with caution and may not reflect the national distribution.

The custom assay was proposed and developed before the commercial assay became available for purchase within the project. Therefore, the commercial assay was introduced at a later stage as a complementary method to support validation and comparison under real laboratory conditions. The findings demonstrate the feasibility and analytical accuracy of SMA screening in this setting, with sensitivity and specificity consistent with international reports (>95–99%), although these estimates should be interpreted cautiously given the small sample size [8,32]. While the commercial assay used in this study enables extended genotyping (including *SMN2*), its role as a screening tool requires confirmatory testing. In this context, an *SMN1*-focused in-house approach may represent a cost-effective option for primary screening, particularly in resource-limited settings. Beyond some cost considerations, the in-house assay also offered advantages related to compatibility with the existing laboratory infrastructure, protocol flexibility, direct control over assay optimization and validation, reduced dependence on external suppliers, and development of local technical expertise.

Thus, the comparison was not intended to demonstrate the superiority of one assay over the other but rather to assess the feasibility and applicability of both approaches within the existing infrastructure of the HMGL of the IMC.

The implementation of neonatal SMA genetic screening in the Republic of Moldova demonstrates high feasibility and could be integrated efficiently, largely due to the pre-existing infrastructure for phenylketonuria screening and the established workflow for DBS sample collection across maternity hospitals, together with molecular methods implemented locally (qPCR and MLPA) and supported by medical geneticist counseling and interpretation.

The Institute of Mother and Child, the main tertiary maternity center and national center for PKU newborn screening, serves a large and diverse obstetric population from across the country, and its nationally centered DBS collection supports implementation without additional logistical restructuring for the whole country. Early identification enables immediate treatment, which international studies show dramatically reduces mortality and severe disability, especially when therapy is initiated presymptomatically [33,34]. This aligns the Republic of Moldova with settings where SMA newborn screening has been implemented or is being introduced [35,36,37,38,39,40,41,42,43]. From a public health perspective, early detection is cost-effective: long-term healthcare expenditures are reduced by avoiding prolonged hospitalizations and late-stage interventions [43,44]. Integration into existing neonatal screening programs would enable nationwide SMA screening and provide a platform for expanding testing to other severe genetic disorders using the same DBS samples.

Considering the limited number of samples analyzed and the study design, this work should be interpreted as a feasibility and implementation study rather than as a population-based neonatal screening pilot. Accordingly, it was not intended to estimate SMA prevalence or population-level detection rates in the Republic of Moldova. Its main objective was to improve the quality of SMA diagnosis by developing and implementing molecular genetic tests adapted to the existing infrastructure. In parallel, the study assessed the practical applicability of dried-blood-spot-based SMA testing within the institutional workflow and evaluated the feasibility of integrating genetic testing into neonatal screening procedures. It also supported the establishment of clinical, laboratory, and referral protocols required for future expansion. Overall, these findings provide a methodological and organizational foundation for a subsequent nationwide, population-based screening program.

However, successful nationwide implementation requires system strengthening, including expansion of trained personnel, laboratory capacity, standardized protocols, compliance with data protection and ethical regulations, and effective public awareness and communication to support informed participation and timely follow-up. Thus, the initial steps in the Republic of Moldova focused on the development and implementation of a national clinical protocol for SMA, which includes a clearly defined workflow for genetic diagnosis and emphasizes the critical importance of presymptomatic identification [24]. In parallel, a regulatory document describing the neonatal genetic screening workflow (logistics and methods) was developed and formally registered with the State Agency for Intellectual Property (copyright No. OȘm 8347, 21 October 2025) [45], supporting the operational implementation of the screening program. These initiatives represent foundational milestones toward integrating into routine neonatal screening a genetic disorder for which no biochemical biomarker is available.

According to the revised Wilson and Jungner criteria and their subsequent updates [46,47], the availability of an accepted intervention is a fundamental prerequisite for the implementation of any neonatal screening program. Such an intervention may include treatment, prevention, or genetic counseling and should form part of a coherent management strategy, although it does not necessarily imply proven efficacy. Equally important is the formal registration and regulatory approval of the corresponding medicinal product by the National Medicines and Medical Devices Authority, a step that is particularly challenging in non-EU countries. In the Republic of Moldova, risdiplam (Evrysdi^®^) was registered in 2022 for the treatment of 5q spinal muscular atrophy in patients aged two months and older, with a clinical diagnosis of SMA type 1, type 2, or type 3, or with one to four copies of the SMN2 gene [48]. The availability of this disease-modifying therapy represents a critical enabling factor for the national implementation of SMA neonatal screening, ensuring that treatment can be initiated before the onset of irreversible clinical manifestations.

Overall, this feasibility study provides relevant and strategic steps for implementing neonatal SMA screening in a routine mode, thus offering a strategic investment that enhances equity in access to early genetic diagnosis and aligns Moldova with global trends in advanced newborn screening.

## 5. Conclusions

This pilot study demonstrates the feasibility of implementing neonatal genetic screening for spinal muscular atrophy in the Republic of Moldova, supported by existing dried blood spot infrastructure, established sample logistics, and locally available molecular diagnostic methods (qPCR and MLPA). The results further indicate that both custom-developed and infrastructure-compatible commercial assays may support implementation in this setting, provided that sustained institutional and governmental support is ensured. Among 346 screened newborns, two SMA cases were identified and genetically confirmed. The observed diagnostic performance was consistent with international standards, supporting reliable early detection and the possibility of timely therapeutic intervention to improve survival and motor outcomes, in line with evidence from countries where SMA screening has been implemented.

From a public health perspective, the program has the potential to be cost-effective by reducing long-term healthcare costs associated with severe disability, prolonged hospitalization, and late-stage interventions. Integration into the national newborn screening framework is feasible, as SMA testing can be incorporated into existing PKU screening workflows. Moreover, the National Program on Rare Diseases for 2024–2028 foresees the expansion of newborn screening in the Republic of Moldova. In this context, procurement has been initiated for a tandem mass spectrometer to enable screening for PKU and up to 20 inborn errors of metabolism, as well as a new fluorometer to support future screening for cystic fibrosis, congenital hypothyroidism, and galactosemia. These developments indicate that SMA screening could be integrated as part of a broader national strategy for expanding NBS.

However, nationwide implementation will require strengthened workforce capacity, laboratory infrastructure, standardized national protocols, coordination, and compliance with NBS requirements. Overall, these findings support the expansion of SMA newborn screening to all newborns in the Republic of Moldova. Importantly, the transition from an absence of SMA screening protocols and molecular diagnosis to the successful implementation of a DBS-based qPCR/MLPA workflow demonstrates the operational feasibility of early detection in a real-world, lower-resource setting based on research projects. These findings provide policy-relevant evidence for scaling up the program, supporting the development of national protocols, and positioning SMA screening as a viable addition to the expanding national NBS program.

## Figures and Tables

**Table 1:** Overview of SMA genetic screening results obtained using both custom and commercial tests.

Test Type	No. of Samples	Positive/Negative Results	Interpretation	Analytical Sensitivity	Clinical Sensitivity	Specificity	Internal Validation
**Customized** **HMGL**	346	2/344	2 positive for SMA; 344 negative	100%	95%	100%	*ALB* exon 12—346 of 346 samples
**Commercial (Neo** **Nat SCID-** **SMA)**	75	1/74	1 positive for SMA; 74 negative	100%	95%	100%	*β*-globin—75 of 75 samples

**Table 2:** Comparative cost and workflow efficiency based on maximum 96-well plate utilization.

Parameter	Custom Test (HMGL)	Commercial Test (NeoNat SCID-SMA)
**Plate format**	96-well plate	96-well plate
**Assay format**	3 wells/sample: 2 × *SMN1* + 1 × *ALB*	1 well/sample, multiplex format
**Fixed control/calibrator wells**	Positive, negative, and no-template controls	Manufacturer-defined calibrators and controls
**Maximum samples per** **96-well plate**	32 samples *	Up to 75 samples
**Total cost per full plate/run**	~EUR 155	~EUR 381
**Estimated cost per sample at maximum capacity**	~EUR 5.00–5.20	~EUR 5.10
**Total workflow TAT**	~4 h (pre-analytical sample registration, DBS DNA extraction, real-time PCR amplification, and result interpretation)	~2 h (DNA extraction, amplification, and result analysis; excluding pre-analytical sample handling/reporting)
**Sensitivity/specificity**	100%/100%	100%/100%
**Platform used**	7500 Applied Biosystems	7500 Applied Biosystems
**Software/chemistry**	7500 Software v2.3, SYBR Green Reagents/melt curve	7500 Software v2.3, TaqMan Reagents

**Note.** TAT, turnaround time. For the HMGL method, TAT refers to the complete analytical workflow from DBS DNA extraction to result interpretation. For the commercial kit, TAT should be reported according to the manufacturer’s protocol or measured under the same local workflow conditions. * For the HMGL method, maximum sample capacity depends on the number of wells allocated to positive, negative, and no-template controls.

## Data Availability

The original contributions presented in this study are included in the article. Additional data may be available upon request, where ethically and legally permissible.

## Supplementary Materials

The following supporting information can be downloaded at: https://www.mdpi.com/article/10.3390/ijns12020038/s1; Figure S1: Template letter informing parents of the rejection of the neonatal screening sample due to technical reasons; Figure S2: Template letter informing parents of the absence or non-identification of the neonatal screening sample; Figure S3: Template letter notifying parents of a positive neonatal screening result for spinal muscular atrophy; Form S1: SMA screening and genetic diagnosis registration form.

## Abbreviations

The following abbreviations are used in this manuscript:

SMA	Spinal Muscular Atrophy
NBS	Newborn Screening
MLPA	Multiplex Ligation-dependent Probe Amplification
qPCR	Real-Time Polymerase Chain Reaction
DBS	Dried Blood Spot
OMIM	Online Mendelian Inheritance in Man
PKU	Phenylketonuria
*SMN1*	Survival Motor Neuron 1 (gene)
*SMN2*	Survival Motor Neuron 2 (gene)
*ALB*	Albumin (gene)
*NAIP*	Neuronal Apoptosis Inhibitory Protein
*β-Globin*	Beta-Globin (gene)
CNV	Copy Number Variation
EDTA	Ethylenediaminetetraacetic Acid
SYBR	SYBR Green (fluorescent dye used in PCR)
HMGL	Human Molecular Genetics Laboratory
IMC	Institute of Mother and Child
PhD	Doctor of Philosophy
PCN	National Clinical Protocol
SCREENGEN	Genomic medicine and metabolomic research in the service of genetic disease prevention for healthy generations (project)
DNA	Deoxyribonucleic Acid
UMPh	University of Medicine and Pharmacy
dsDNA HS	Double-stranded DNA High Sensitivity
ng/μL	Nanograms per microliter
EpiInfo (StatCalc)	Epidemiological software (StatCalc module)
ΔΔCt	Delta Delta Cycle Threshold
WMA	World Medical Association (Declaration of Helsinki)
HRM	High-Resolution Melting
NeoNat	Neonatology
SCID	Severe Combined Immunodeficiency
