# Supplementary material for: Newborn Screening for Spinal Muscular Atrophy in the Republic of Moldova: A Feasibility Study and First Steps
Source: Int J Neonatal Screen. 2026 May 28;12(2):38. doi: 10.3390/ijns12020038 (PMC13299184; doi:10.3390/ijns12020038)
Supplement: Supplementary file 1 [file IJNS-12-00038-s001.zip › IJNS-4239086-supplementary files/Form F1. SMA_Screening_and_Genetic_Diagnosis_Registration_Form_EN.pdf]

Form for Screening and Molecular Genetic Diagnosis
of Spinal Muscular Atrophy (SMA) in Newborns

A. General Participant Information

Participant's Last Name/First Name ______________________________ Sex (F/M) _______
Date of Birth ______________ Date of Sample Collection ___________ Birth Weight (g) ____
Record No./Department ________/______ Apgar Score at Birth _________________________
Address ____

B. Neonatal Screening

Registration Code for Neonatal Screening ____
Screening Result (qPCR deletion of SMN1 exon 7) (Positive/Negative)__________________

N.B. In case of a positive result, Section C must be completed.

C. Diagnosis (to be performed and completed in case of a positive neonatal screening result)

Registration Code for SMA Diagnosis ____
Diagnostic Result (MLPA, kit P021) (SMA positive/SMA negative)_____________________

Gene	Number of Copies (digits)	Code
SMN1		
SMN2		

Result codes:
1 - Homozygous normal (wild type)
2 - Homozygous deletion
3 - Heterozygous deletion
4 - Heterozygous duplication

Date _____ Signature ______________
