# Supplementary figures and images for: Newborn Screening for Spinal Muscular Atrophy in the Republic of Moldova: A Feasibility Study and First Steps
Source: Int J Neonatal Screen. 2026 May 28;12(2):38. doi: 10.3390/ijns12020038 (PMC13299184; doi:10.3390/ijns12020038)

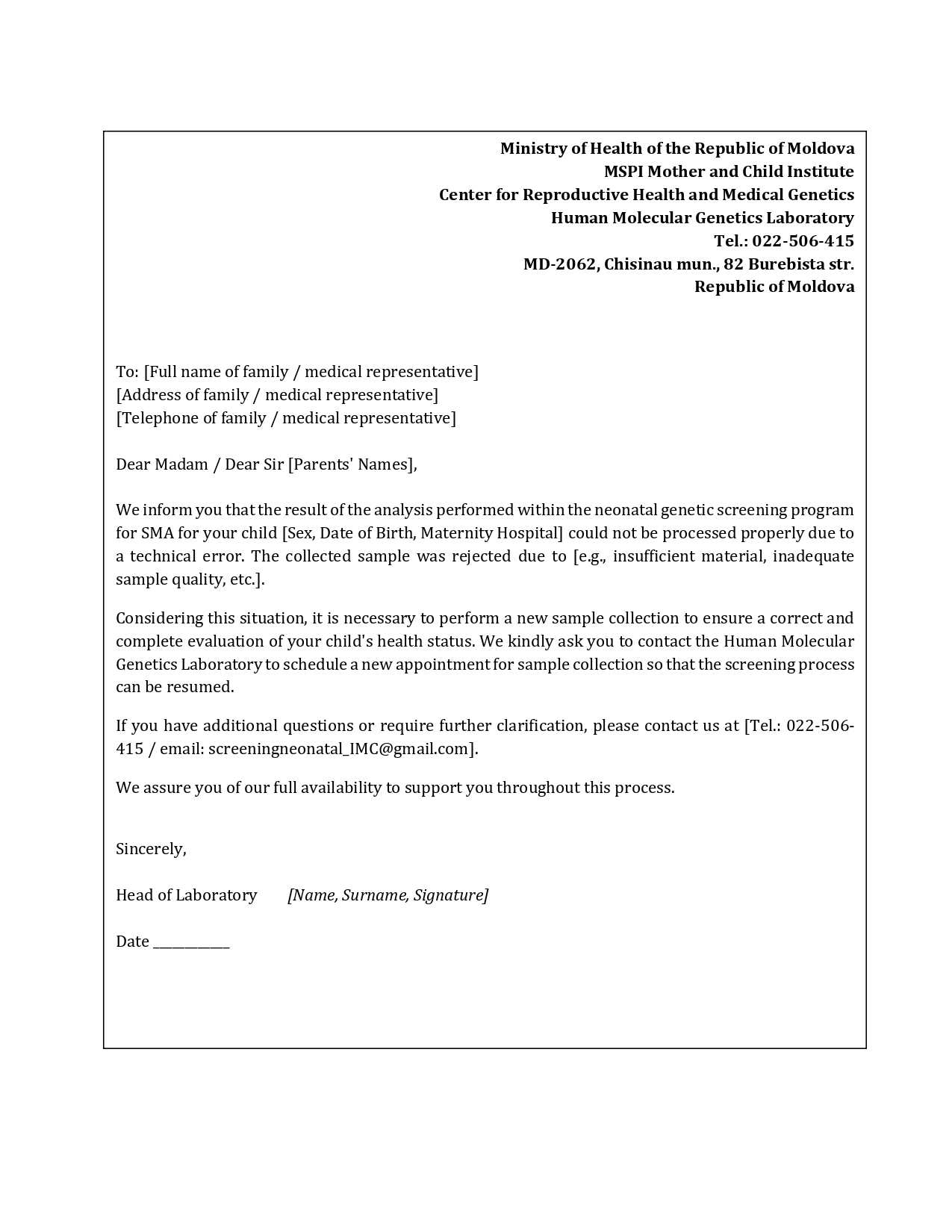

Supplement: Supplementary file 1 [file IJNS-12-00038-s001.zip › IJNS-4239086-supplementary files/Figure S1 Template letter informing parents of the rejection of the neonatal screening sample.jpg]

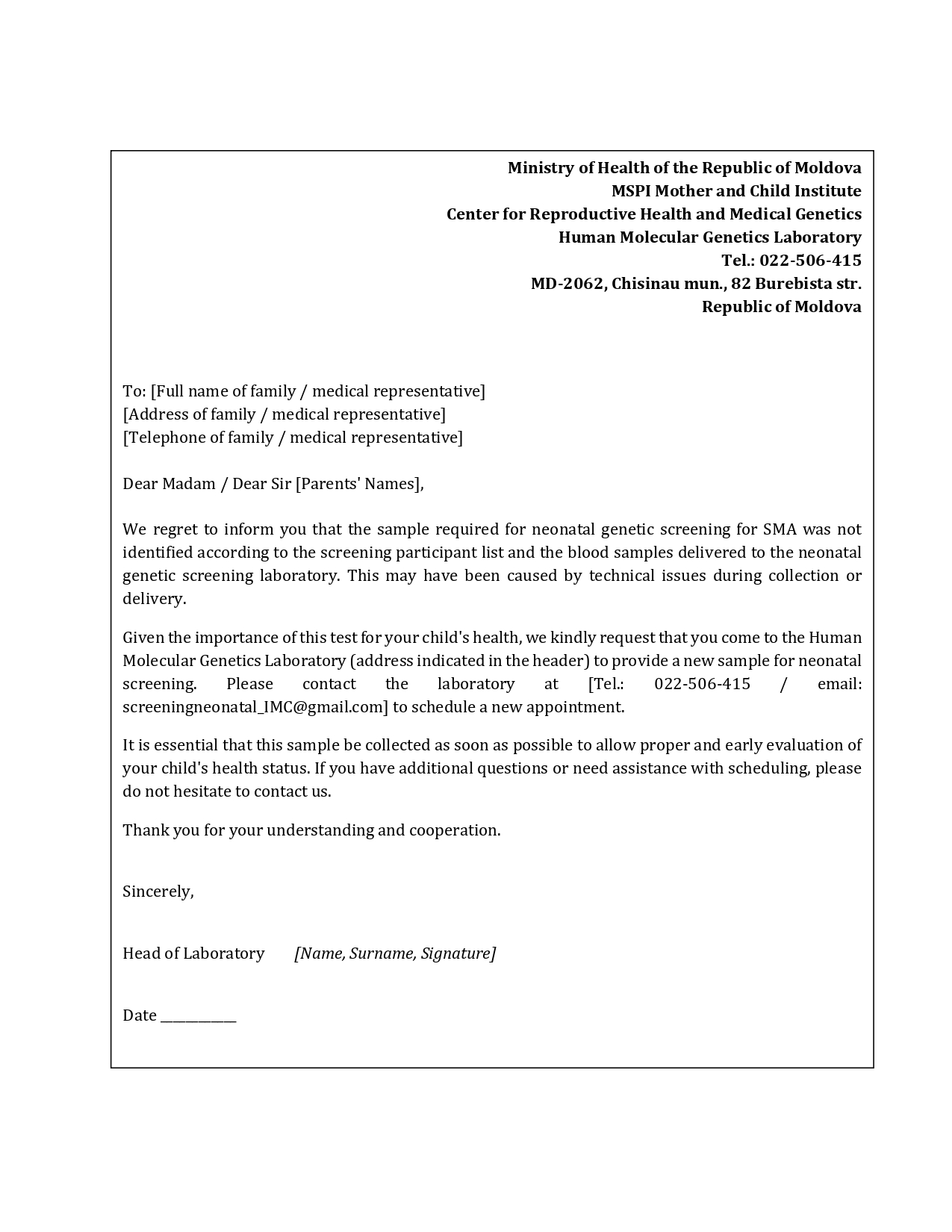

Supplement: Supplementary file 1 [file IJNS-12-00038-s001.zip › IJNS-4239086-supplementary files/Figure S2 Template letter informing parents of the absence or non-identification of the sample.jpg]

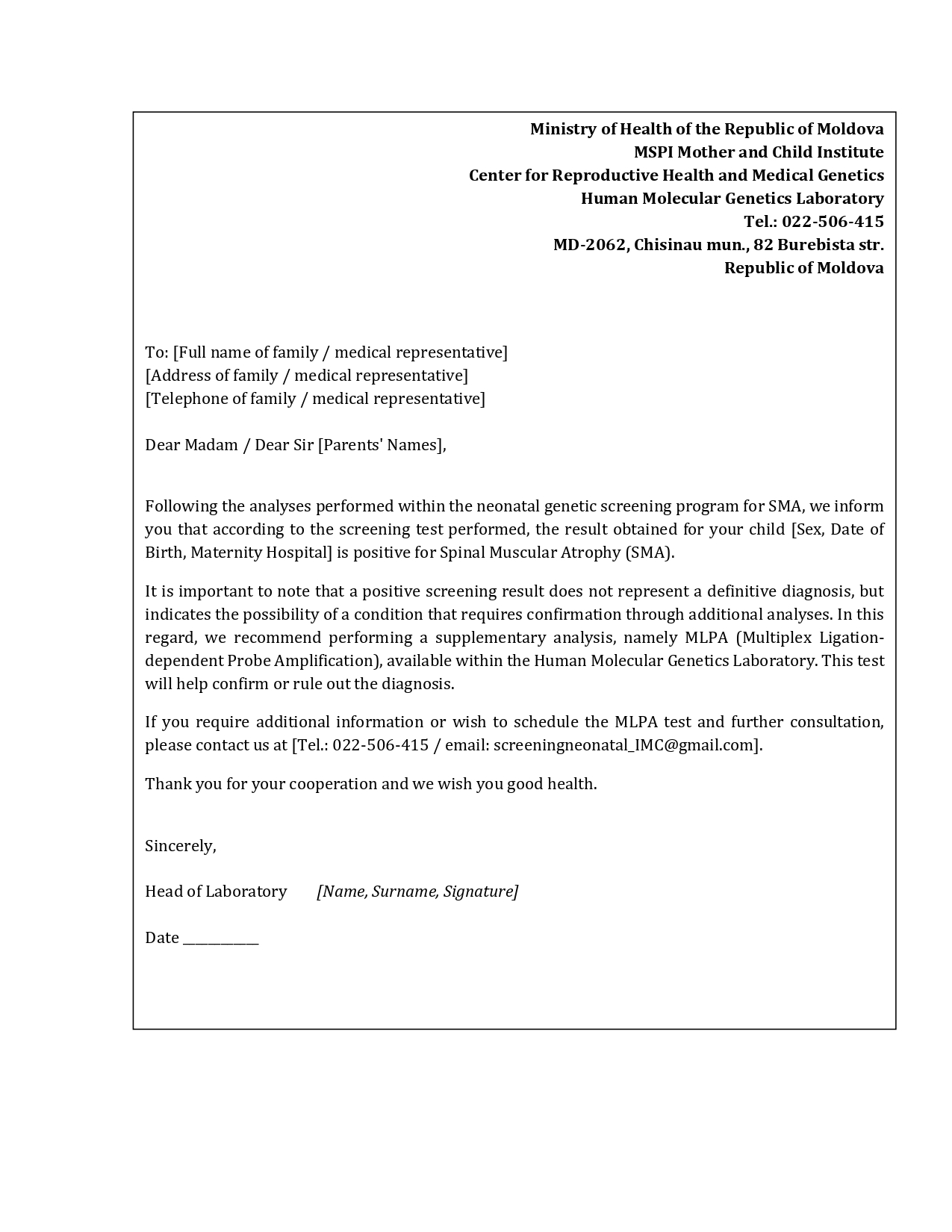

Supplement: Supplementary file 1 [file IJNS-12-00038-s001.zip › IJNS-4239086-supplementary files/Figure S3 Template letter notifying parents of a positive neonatal screening result for SMA.jpg]
